# COVID-19 among Healthcare Workers in the University Clinical Hospital in Wroclaw, Poland

**DOI:** 10.3390/ijerph18115600

**Published:** 2021-05-24

**Authors:** Jarosław Drobnik, Robert Susło, Piotr Pobrotyn, Ewa Fabich, Violetta Magiera, Dorota Diakowska, Izabella Uchmanowicz

**Affiliations:** 1 Gerontology Unit, Public Health Department, Wroclaw Medical University, 51-618 Wroclaw, Poland; jaroslaw.drobnik@umed.wroc.pl (J.D.); robert.suslo@umed.wroc.pl (R.S.); 2 Clinical Hospital Management, Wroclaw Medical University, 50-529 Wroclaw, Poland; piotr@citodent.pl (P.P.); efabich@usk.wroc.pl (E.F.); viola.magiera@gmail.com (V.M.); 3 Department of Nervous System Diseases, Faculty of Health Sciences, Wroclaw Medical University, 51-618 Wroclaw, Poland; dorota.diakowska@umed.wroc.pl; 4 Department of Clinical Nursing, Faculty of Health Sciences, Wroclaw Medical University, 51-618 Wroclaw, Poland

**Keywords:** COVID-19, healthcare workers, Polish hospitals

## Abstract

Efforts to limit severe acute respiratory syndrome coronavirus 2 (SARS-CoV-2) infections among hospital healthcare staff are crucial for controlling the Coronavirus Disease 19 (COVID-19) pandemics. The study aimed to explore the prevalence and clinical presentations of COVID-19 in healthcare workers (HCWs) at the University Clinical Hospital (UCH) in Wroclaw with 1677 beds. The retrospective study was performed in 2020 using a self-derived structured questionnaire in a sample of HCWs who were diagnosed with SARS-CoV-2 infection confirmed using a PCR double gene test and consented to be enrolled into the study. The significance level for all statistical tests was set to 0.05. The study showed that of the 4998 hospital workers, among 356 cases reported as COVID-19 infected, 70 consented to take part in the survey: nurses (48.5%), doctors (17.1%), HCWs with patient contact (10.0%), other HCWs without patient contact (7.1%), and cleaning personnel (5.7%). HCWs reported concurrent diseases such as hypertension (17.1%), bronchial asthma (5.7%), and diabetes (5.7%). Failure to keep 2 m distancing during contact (65.5%) and close contact with the infected person 14 days before the onset of symptoms or collection of biological material (58.6%) were identified as the increased risks of infection. A large part of infections in hospital healthcare staff were symptomatic (42.9%). The first symptoms of COVID-19 were general weakness (42.9%), poor mental condition (41.4%), and muscle pain (32.9%); whereas in the later stages of the illness, general weakness (38.6%), coughing (34.3%), lack of appetite (31.4%), and loss of taste (31.4%) were observed. In about 30% of the infected HCWs, there was no COVID-19 symptoms whatsoever. The vast majority of the patients were treated at home (85.7%). In conclusion, the majority of the SARS-CoV-2 infections in the hospital HCWs were asymptomatic or mildly symptomatic. Therefore, successful limitation of COVID-19 infection spread at hospitals requires a close attention to future cross-infections.

## 1. Introduction

Severe acute respiratory syndrome coronavirus 2 (SARS-CoV-2) is a new strain of the coronavirus that has been first detected in Wuhan city (Hubei province, China (PRC) with a population of more than 11 million inhabitants. The outbreak began as pneumonia of unknown etiology in late December 2019. On 11 February 2020, an isolated pathogen causing this human infection was named by The International Committee on the Taxonomy of Viruses (ICTV) as the virus SARS-CoV-2. Finally, the World Health Organization (WHO) has announced that the disease caused by this virus is coronavirus disease 2019 (COVID-19) [1].

Given a massive burst of coronavirus disease worldwide, the WHO has updated recommendations for the classification of diseases and related health problems (ICD-10) and introduced two new codes for laboratory-confirmed cases of COVID-19 as well as suspected or confirmed SARS-CoV-2 infection [2].

In Poland, the general rules defining SARS-CoV-2 infection prevention measures, cases classification and reporting, diagnostics, and treatment were set by the central government [3] and its agencies [4]. They were further developed and then implemented locally by district experts and physicians specializing in epidemiology and infectious diseases, hospital infections control teams, as well as by particular members of medical staff [5]. Since SARS-CoV-2 infection can spread rapidly, healthcare workers (HCWs) responsible for treating COVID-19-positive patients are at greater risk of this infection. 

The present work shows the spread of infections among the University Clinical Hospital in Wroclaw, Poland (UCH) healthcare employees. The hospital is a 1677-bed facility and employs 4998 staff members. During the period under review from March to April 2020, UCH hospital had continued to use two-gene polymerase chain reaction (PCR) tests for SARS-CoV-2 and implemented in parallel three primary infection-control strategies for identification of cases of COVID-19 among UCH medical personnel. The first strategy was designed for UCH staff members who had developed symptoms of suspected SARS-CoV-2 infection. In that case, SARS-CoV-2 testing was performed immediately to recognize as early as possible transmission paths by identifying close contacts with other UCH staff or residents. The second strategy was implemented when there was suspected close contact of asymptomatic UCH personnel with a person infected with SARS-CoV-2 without adequate protection, in order to shorten the quarantine period from the standard (initially 14-day, then 10-day) to 7-day and thereby reduce the period of removal from work of deficient medical personnel, which is in agreement with the Voivodship Sanitary and Epidemiological Station (Sanepid), staff examinations were carried out on the 7th day after the last close contact. A negative result of the test made it possible to apply to Sanepid to shorten the quarantine and allow the given UCH staff member to come back work. The third strategy was applied when it was not possible to exclude close contacts without adequate protection of many UCH personnel with a person infected with SARS-CoV-2, or there was an outbreak of SARS-CoV-2 infections with an undetermined starting point in a UCH unit, and there was a need to exclude that there is an asymptomatic person among USK personnel who could be the source of infection, a cross-sectional study for SARS-CoV-2 infection covering all UCH personnel of the unit was carried out.

The time distribution of the registered SARS-CoV-2 infection cases among the UCH personnel in the year 2020 (Table 1) was similar as in the general Lower Silesia region population [6], with the recognizable peaks during the spring and autumn months, which are commonly referred to as “waves” of COVID-19 epidemics.

**Figure 1:**
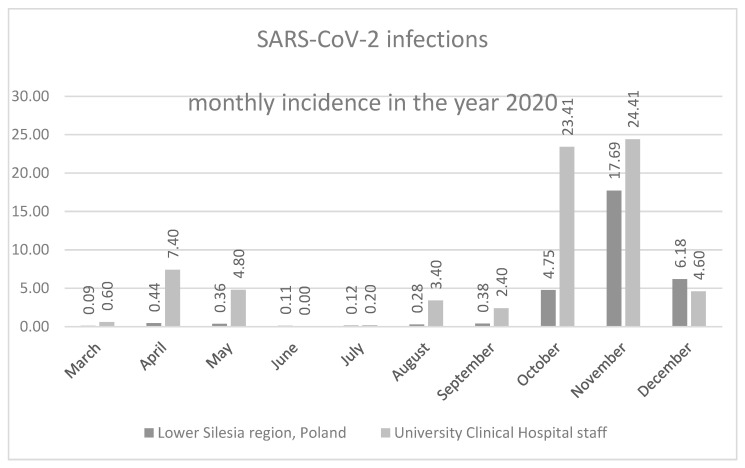
Monthly incidence of new SARS-CoV-2 infections among the Lower Silesia region population and among the University Clinical Hospital in Wrocław staff in the year 2020, per 1000 population.

Interestingly, the SARS-CoV-2 incidence in the year 2020 was significantly higher among the 4.998 UCH personnel than in the general Lower Silesia population of 2.9 million, with the single exception of December 2020 (Figure 1).

The study aimed to explore the prevalence and clinical presentations of COVID-19 among hospital care personnel at the University Hospital in Wroclaw, Poland.

## 2. Materials and Methods

### 2.1. Setting, Design, and Participants

The study was performed among the staff of University Clinical Hospital (UCH) from March to October 2020, who were recruited retrospectively. The number of HCWs, according to their profession and department, during the study period was obtained from the human resources department. This study used an observational and cross-sectional designs; therefore, the Strengthening of the Reporting of Observational Studies in Epidemiology (STROBE) guidelines were followed.

### 2.2. Qualification Procedure

The inclusion criteria were identification of COVID-19 (RT-PCR) positive test and informed consent to participate in the study. The exclusion criterion was no consent to take part in the study.

### 2.3. Research Tools

The study used a self-developed by authors structured questionnaire targeting data concerning exposure, epidemiologic, clinical, and demographic parameters. The anonymous survey has been conducted among the HCWs who between 1 January 2020 and 15 October 2020 had been diagnosed with SARS-CoV-2 infection. The participants have been informed on their right to abstain from participation. The questionnaire includes questions pertaining to gender; age; characteristics of the study participant’s place of accommodation; place where the participant has spent the time of obligatory isolation and treatment; the characteristics of the COVID-19 symptoms present at the start and during the period of illness; time of first COVID-19 symptoms manifestation and perceived level of their severity; date of the first positive result of the SARS-CoV-2 test and the time of the first negative result of that test afterwards; as well as the characteristics of known to the study participant close contacts posing risk of SARS-CoV-2 transmission from people known as already infected, which possibly led to the participant’s SARS-CoV-2 infection; and the safety precautions and measures applied by the participant at the time of those contacts. The results of the questionnaire study were merged with data derived from analysis of hospital medical documents, including epidemiology investigation reports, medical files, and laboratory tests results, aiming at identification of the characteristics of medical workers’ exposure to the risk of acquiring the SARS-CoV-2 infection and ways of spreading this infection inside the hospital.

### 2.4. Ethical Considerations

All respondents received information about the study procedure and research aims and provided informed consent to participate. Full anonymity was guaranteed. The study was performed in accordance with Declaration of Helsinki and approved by the local bioethics committee of University Clinical Hospital at Wroclaw (permission no. 732/2020).

### 2.5. Statistical Analysis

Descriptive data were presented as the number of observations and percentages. The chi-squared test with Yates’ correction for 2 × 2 tables was used to compare qualitative variables among the groups. For the low number of counts in the contingency tables, the Fisher’s exact test was used instead. The Mann–Whitney test was used to compare quantitative variables between two groups, while the Kruskal–Wallis test with post hoc multiple comparisons based on the Dunn statistics was used for more than two groups. Single-factor and multi-factor analyses to evaluate the effects of risk factor variables was performed using the binary logistic regression (BLR) model. The resulting values were presented as odds ratio (OR) parameters with a 95% confidence interval (CI). A significance level of 0.05 was assumed in the analysis; therefore, all *p*-values below 0.05 were interpreted as indicating significant dependencies. Statistical analyses were performed using R software v. 4.0.3 [7].

## 3. Results

The structure of the healthcare employees in the study period is presented in Table 2. Of the 4998 workers, 356 were diagnosed with SARS-CoV-2 infection, and 70 of them consented to take part in the survey. The mean age of infected HCWs was 46.8 ± 11.3, and 77.1% were women (Table 3). Table 3 provides a description of demographic, occupational and clinical characteristics of the sample. Around 81.4% of medical staff lived in the city. The infected medical staff in the studying sample included nurses (48.5%), doctors (17.1%), other medical professionals being with in contact with patients (10.0%), other medical staff without contact with patients (7.1%), and hospital cleaning personnel (5.7%). Samples of medical staff had comorbidity: hypertension (17.1%), bronchial asthma (5.7%), and diabetes (5.7%).

Retrospective analysis of in-hospital infections of COVID-19 identified two risk factors of the virus transmission, which was failure to keep 2 m distance during contact (65.5%) and close contact with the infected person 14 days before the identification of symptoms or collection of biological material (58.6%) (Table 4).

Furthermore, the univariable and multivariable linear regression analyses was run to identified risk factors associated with COVID-19 infection. Any of the study parameters were found to be the independent risk factors of COVID-19 infection in hospital healthcare professionals (Table 5). As shown in Table 5, a large part of infections in medical staff was symptomatic (42.9%); however, there were also cases of HCWs categorized as asymptomatic (28.6%) and mildly symptomatic (27.1%) at the time of testing. The majority of the patients were treated at home (85.7%); only two patients were treated in the isolation ward. The first symptoms of COVID-19 were general weakness (42.9%), poor mental condition (41.4%), and muscle pain (32.9%). The following symptoms were successive: general weakness (38.6%), coughing (34.3%), lack of appetite (31.4%), and loss of taste (31.4%). About 30% of hospital staff members reported no COVID-19 symptoms (Table 5).

### 3.1. Factors Predicting Symptomatic or Severe COVID-19 Disease

Logistic regression modeling was performed to identify factors predicting symptomatic or severe COVID-19 in medical staff (*p* < 0.05). We found that the factor of male sex was associated with the significant decreased risk of a symptomatic or severe form of COVID-19 (OR = 0.25; 95% CI). The other predictor was contact without a mask that was associated with the increased risk of the symptomatic or severe COVID-19 (OR = 6.389; 95% CI). For the predictor of contact with the mask, there was also the increased risk of symptomatic or severe forms of the COVID-19 disease (OR = 5897; 95% CI). Finally, close contacts (below 2 m) also increased the odds for asymptomatic or severe COVID-19 (OR= 4533; 95% CI: (Table 6).

Then, the multivariate logistic regression was used to identify (*p* < 0.05) independent predictors of symptomatic or severe COVID-19 infections. We found that the independent predictor was male sex (OR = 0.058, 95% CI), which reduced the odds of symptomatic or severe COVID-19. In contrast, the physical contact independently predicted the increased risk of symptoms or severe COVID-19 (OR = 37.132, 95% CI) (Table 6).

### 3.2. Analyses of the Risk Factors of Scarcely Symptomatic, Symptomatic or Severe COVID-19

We ran logistic regression analyses to distinguish significant (*p* < 0.05) predictors of the risks of scarcely symptomatic, symptomatic, or severe COVID-19 infections. The factors of close contacts (OR = 5.227; 95% CI), and contact at a distance below 2 m (OR = 4.792; 95% CI) were associated with the higher risks of scarcely symptomatic, symptomatic, or severe COVID-19 infections (see Table 7). The multivariable logistic regression showed that none of the factors analyzed was the independent predictor of the risks of a scant-symptomatic, symptomatic, or severe COVID-19 (*p* > 0.05) (Table 7).

## 4. Discussion

According to the Ministry of Health in Poland, from the beginning of the COVID-19 epidemic (4 March 2020) to 9 September 2020, coronavirus infections have been confirmed in Polish medical facilities: 1389 doctors, 3276 nurses, 268 midwives, 103 laboratory diagnosticians, 113 dentists, 83 pharmacists, and 312 paramedics. Moreover, SARS-CoV-2 infection has contributed to the deaths of eight doctors, six nurses, and one paramedic [8].

This study analyzed the epidemiology of SARS-CoV-2 infection among healthcare staff of a University Hospital in Wroclaw during the peak of the COVID-19 epidemic in Poland. The hospital is the largest facility in the region, providing medical services to about 100,000 hospitalized patients yearly and about 110 outpatients treated in Emergency and Admissions Ward every day [9]. This implicates that infection with the SARS-CoV-2 virus there has become a potential threat to the hospital medical staff (4998 workers) at the time of ongoing pandemic despite a variety of measures to limit the risk of the SARS-CoV-2 transmission and cross-contamination among the staff. These included among others attempts at forming a team separation system. Practically, due to the notorious scarcity of the personnel aggravated by the SARS-CoV-2 infections-related absenteeism in Poland, it was only possible for a limited time and in limited hospital units, mainly those that were less loaded with work due to planned hospital admission limitations, as the beds were temporarily re-profiled to suit the needs of COVID-19 patients. The hospital staff working hours are typically divided either into 8 or 12 h long shifts (in case of physicians on working weekdays and all other hospital staff) or into 12, 16, or 24 h long on-duty hours (mostly physicians after routine working hours, on weekends and other free-of-work days). Above all, the University Clinical Hospital provided all the staff members with surgical facemasks and demanded from the staff to wear the masks all the time while at the hospital; additional hand rub dispensers were distributed for personal use to be placed directly at all workplaces as well. The staff members—while working at the hospital locations where the SARS-CoV-2 infected patients, or patients suspected of such infection, were treated—were additionally obligatorily equipped with FFP2/FFP3 facemasks, eye protection (goggles/shield), disposable overalls/reinforced waterproof gowns, and double sets of disposable medical gloves. In addition, all patients were required to wear face masks all the time while at the hospital and any room at the hospital where a SARS-CoV-2 infected patient, or a patient suspected of such infection, was residing, was transported without being isolated with a facemask, disposable gown, and gloves, or underwent a medical procedure, was subsequently subjected to additional comprehensive thorough cleaning with antivirus solutions, especially including all contact surfaces and equipment, and biocide dry-fog treatment of the room of the duration indicated in the manufacturer’s directions.

Nevertheless, staff of medical facilities in Poland worked during the COVID-19 pandemic under increased psychological pressure, which resulted not only from demands of treating patients ill with unknown yet disease and work overload in a dynamically changing environment but also from the looming perspective of subsequent medico-legal problems. Theoretically, there is a general “good Samaritan’s clause” in art. 24 of the special Polish legal regulation for the time of COVID-19 [10] that is currently in operation. It was introduced to exculpate medical staff from criminal responsibility for possible mistakes they may have made in COVID-19 treatment during the epidemic. Highly unfortunately, in practice, it is of low importance to the medical staff, as it does not alleviate their criminal law responsibility in case of any other medical mistakes, including those made while treating conditions other than COVID-19 but precipitated by the extraordinary conditions and limitations during the coronavirus pandemics. Moreover, there is no legal shield of any kind in case of medical error during the COVID-19 epidemic-related civil lawsuits against medical professionals or medical facilities. The practical validity of the COVID-19-related legal alleviations was not tested yet, as there were no legal lawsuits against the UCH related to COVID-19 until the time of the study.

The incubation period of the virus is relatively long compared to other viruses such as SARS and Middle East Respiratory Syndrome (MERS) [11], which may be an additional exposure factor for medical staff to get infected. Additionally, healthcare workers could be in the chains of in-hospital transmission [12]. The clinical spectrum of COVID-19 varies from asymptomatic infections to severe respiratory symptoms and death [13]. The majority of SARS-CoV-2 infections among healthcare workers were symptomatic (71.4%), although a large proportion of medical staff was asymptomatic (28.6%). This certainly had contributed to an ongoing SARS-CoV-2 transmission to patients and other HCWs during the study period. It is worth mentioning that a large amount of infected employees were not staff devoted to working with COVID-19-positive patients. The infected workers included nurses (48.5%), doctors (17.1%), other medical staff with patient contact (10.0%), HCWs with no patient contact (7.1%), and cleaning personnel (5.7%). HCWs reported the presence of common comorbidities: hypertension (17.1%), bronchial asthma (5.7%), and diabetes (5.7%). 

The analysis of early transmission dynamics in Wuhan showed that asymptomatic cases of SARS infection were confirmed only in 0.9% of HCWs [14,15]. Therefore, identification of asymptomatic cases in HCWs may be crucial for preventing cross-infection. The present study showed that in-hospital infection transmission had occurred throughout close contacts (physical distancing less than 2 m) (65.5%) with the infected person. This suggests that HCWs who subsequently tested positive did not comply with the hospital protection restrictions in terms of respiratory hygiene and screening policies for any symptoms of upper respiratory tract infection [16]. However, in the first period of the epidemic in Poland in April 2020, only 70 of COVID-19 infections were recorded among 4998 of the hospital in the 1677-bed facility. Currently, the extent of COVID-19 transmission and its relevant infection risk factors in healthcare settings remain unclear [17]. For instance, the Chinese studies show that 2055 laboratory-confirmed cases were reported among HCWs from 476 hospitals. In these studies, most infections among HCWs were suspected to take place in the outside of healthcare settings [14,15]. In another study on COVID-19 infections in HCWs in the Netherlands, in March 2020, 45 (4%) of 1097 HCWs from nine hospitals tested positive for COVID-19 [18]. The investigated HCU hospital had 70 people tested positive, which indicated the infection rate of 1.4%, which was higher as compared to the infection rates of 0.18% in Wuhan [14] and 1.1% in Tongji hospital [15]. Due to the prevalence of COVID-19 among hospital staff, regular testing of hospital staff has been introduced.

During the period under review, two-gene PCR tests for SARS-CoV-2 infections were used at the UCH facility, and the three main strategies for testing UCH personnel described in the introduction were applied in parallel. The European Centre for Disease Prevention and Control (ECDC) is currently in its latest COVID-19 testing strategy paper of September 2020. It recommends periodic testing of healthcare professionals, regardless of symptoms to reduce the transmission of SARS-CoV-2 in medical facilities. The ECDC recommends regular screening of healthcare professionals (e.g., every week or every two weeks), along with their personal protective equipment and requires the daily monitoring of symptoms among medical staff and cessation of work by an employee who develops COVID-19 symptoms. The ECDC bases its recommendations on the modeling studies that have demonstrated the reduction of SARS-CoV-2 transmission by 23% through the routine molecular testing for COVID-19 among healthcare professionals with the assumptions that test results are available within 24 h. Such an infection-control strategy is recommended by the ECDC in regions where there is the local transmission of SARS-CoV-2, regardless of its severity [19]. At the University Clinical Hospital in Wrocław, the regular staff screening system for SARS-CoV-2 is as often as every one or two weeks, which never encompassed all staff members. Such a screening system, and for a limited time only, involved only the staff working at the locations that were critical from the point of view of keeping the whole hospital functional (e.g., hospital pharmacy, operation theaters, anesthesia and intensive care units), where there was high frequency of contacts with patients of unknown infection status (e.g., admission and emergency area) or where the patients were treated for whom the health complications resulting from acquiring SARS-CoV-2 infections were particularly severe (e.g., hematology oncology and bone marrow transplantation units). However, in the last 3 months of 2020, all willing members of the staff were offered systematic SARS-CoV-2 PCR test screening designed to be performed on the given person every 6 weeks, assuming that the staff of each hospital unit were divided into three groups tested one after another in time distance of 2 weeks, but the testing scheme was abandoned because of changes in reimbursement strategy for such testing and the start of Polish state-coordinated massive vaccinations against COVID-19 available to medical staff.

The prevention measures recommended by the ECDC do not have to be implemented in communities where the virus has been completely eradicated or is only sporadic. In order to control outbreaks and prevent the re-spread of infections, these kind of measures must be in place [19]. However, there is hope for final resolution of the COVID-19 pandemics crisis, especially eliminating the SARS-CoV-2 infections among HCW, resulting from the registration of COVID-19 vaccines. Unfortunately, the efficacy of the voluntary mass COVID-19 vaccinations coordinated by the Polish government [20] may prove significantly limited, as a significant part of the Polish society remains vaccination skeptics [21], and the promotion of vaccination is unsatisfactory [22]. High levels of vaccination hesitancy is common among societies all around the world that belong to different cultural backgrounds such as Germany [23], Spain [24], Israel [25], Jordan [26], and Japan [27].

This study has some limitations. First, the study was performed at one single center. The recall bias of this survey could be a concern, but information collection has taken place recently; therefore, the possibility of recall bias was slight

## 5. Conclusions

The present study investigated risks of infection with SARS-CoV-2 virus and infection-control strategies proposed by the hospital to prevent from high numbers of infections among healthcare staff. Most of the healthcare professionals were asymptomatic or had mild symptoms; therefore, there is the need to pay attention to future cross-infections in the medical facility.

## Figures and Tables

**Table 1:** Monthly number of new SARS-CoV-2 infections among the Lower Silesia region population [6] and among the University Clinical Hospital in Wrocław in the year 2020.

	March	April	May	June	July	August	September	October	November	December	Total Year 2020
Lower Silesia region, Poland general population	274	1289	1031	325	339	801	1104	13,787	51,313	17,913	88,176
University Clinical Hospital staff	3	37	24	0	1	17	12	117	122	23	356

**Table 2:** The structure of the healthcare workers in the study period.

Type of Personnel	Number of Observation (*n*)	Frequency (%)
Board of Directors	12	0.24
Administration	293	5.86
Doctors—academic teachers from WMU *	311	6.22
Doctors—workers outside UCH **	268	5.36
Doctors—residents	499	9.98
Doctors—junior doctors	119	2.38
Doctors—workers from UCH **	357	7.14
Nursing	1478	29.57
Midwifes	256	5.12
Higher-level non-medical personnel	241	4.82
Middle-level medical personnel	389	7.78
Middle-level non-medical personnel	209	4.18
Lower-level medical personnel	440	8.80
Lower-level non-medical personnel	126	2.52
Total:	4998	100.00

* Wroclaw Medical University, ** University Clinical Hospital.

**Table 3:** Demographic, occupational, and clinical characteristics of study group (*n* = 70).

Variables		*n* (%)
Age (years)	Mean ± SD	46.8 ± 11.3
	Median (Q1–Q3)	49 (40–56)
Sex:	Female	54 (77.14)
	Male	15 (21.43)
	Lack of data	1 (1.43)
Place of residence:	City	57 (81.43)
Place of residence: Profession:	Village	12 (17.14)
	Lack of data	1 (1.43)
	Medical doctor	12 (17.14)
Profession: Place of the work (Unit of University Hospital):	Nurse	34 (48.57)
	Midwife	1 (1.43)
	Paramedic	3 (4.29)
	Physioterapist	1 (1.43)
	Medical caretaker	2 (2.86)
	Cleaning personnel	4 (5.71)
	Others, with contact with patients	7 (10.00)
	Others, without contact with patients	5 (7.14)
	Lack of data	1 (1.43)
	Hospital pharmacy	4 (5.71)
Place of the work (Unit of University Hospital): Concurrent diseases *:	Center of Heart Diseases (CHD)	12 (17.14)
	CHD—Department of Cardiac Surgery	3 (4.29)
	CHD—Hemodynamics Laboratory	2 (2.86)
	Central Admission	1 (1.43)
	Department of Trauma and Hand Surgery	1 (1.43)
	Department of Operating Block	1 (1.43)
	Department of Sterilization	2 (2.86)
	Department of Vascular Surgery	2 (2.86)
	Department of General Surgery	1 (1.43)
	Department of Internal Medicine, Pneumology and Allergology	1 (1.43)
	Department of Internal, Occupational Diseases, Hypertension and Clinical Oncology	6 (8.57)
	Department of Gynecology and Obstetrics	1 (1.43)
	Department of Hematology, Blood Neoplasms and Bone Marrow Transplantation	8 (11.43)
	Department of Nephrology and Transplantation Medicine	1 (1.43)
	Department of Pediatric Nephrology	1 (1.43)
	Department of Pediatric Bone Marrow—Transplantation, Oncology and Hematology	3 (4.29)
	Department of Urology	1 (1.43)
	Internal ward	1 (1.43)
	Intensive Care Unit (ICU)	8 (11.43)
	Pediatric Intensive Care Unit (PICU)	1 (1.43)
	Palliative care	2 (2.86)
	Department of Otolaryngology, Head and Neck Surgery	1 (1.43)
	Department of Psychiatry	1 (1.43)
	Emergency	1 (1.43)
	Department of General and Interventional Radiology and Neuroradiology	2 (2.86)
	Lack of data	2 (2.86)
	Hypertension	12 (17.14)
Concurrent diseases *:	Cardiovascular diseases	1 (1.43)
	Bronchial asthma	4 (5.71)
	Other diseases of respiratory tract	1 (1.43)
	Liver diseases	2 (2.86)
	Diabetes	4 (5.71)
	Others	12 (17.14)

*: The percentages do not add to 100 because each of the patients could have a few or not concurrent diseases.

**Table 4:** Risk factors associated with in-hospital infection of COVID-19 observed in study group (*n* = 70).

Variables		*n* (%)
The social meeting >50 people 14 days before symptoms/collection of biological material	No	62 (88.57)
	Yes	4 (5.71)
	Lack of data	4 (5.71)
Close contact with the infected person 14 days before symptoms/collection of biological material	No	17 (24.29)
	Yes	41 (58.57)
	Lack of data	12 (17.14)
Who was the in close contact with? *	Patients	23 (32.86)
	Healthcare personnel	25 (35.71)
	Family member	3 (4.29)
Use of the mask during contact **	No	7 (17.07)
	Not every time ***	1 (2.44)
	Yes	32 (78.05)
	Lack of data	1 (2.44)
Distance 2 m during contact **	No	27 (65.85)
	Yes	9 (21.95)
	Lack of data	5 (12.20)

*: The percentages do not add to 100 because each of the patients could have a few contacts with several of the infected, **: only patients, who had close contact with infected person 14 days before symptoms/collection of biological material, ***: when in contact with patients—yes, when in contact with hospital personnel—no.

**Table 5:** Clinical characteristics of hospital personnel with COVID-19 (*n* = 70).

Variables		*n* (%)
Diagnosis by hospital screening, SARS-CoV-2 RNA detection	No	18 (25.71)
	Yes	49 (70.00)
	Lack of data	3 (4.29)
Course of the disease	Asymptomatic	20 (28.57)
	Mildly symptomatic	19 (27.14)
	Symptomatic	30 (42.86)
	Critical	1 (1.43)
Place of stay during treatment	Hospital	8 (11.43)
	Isolation ward	2 (2.86)
	Place of residence	60 (85.71)
First symptoms *	Elevated body temperature	19 (27.14)
	Fever	10 (14.29)
	Shivers	17 (24.29)
	Coughing	17 (24.29)
	Throat pain	14 (20.00)
	Shortness of breath or difficulty in breathing	7 (10.00)
	General weakness	30 (42.86)
	Bad mental condition	29 (41.43)
	Head pain	18 (25.71)
	Muscle pain	23 (32.86)
	Chest pain	3 (4.29)
	Abdominal pain	8 (11.43)
	Loose stools or diarrhea	11 (15.71)
	Lack of appetite	14 (20.00)
	Nausea	7 (10.00)
	Loss of smell	15.21.43)
	Loss of taste	17 (24.29)
	Others	5 (7.14)
	Lack of symptoms	20 (28.57)
Next symptoms *	Elevated body temperature	10 (14.29)
	Fever	12 (17.14)
	Shivers	11 (15.71)
	Coughing	24 (34.29)
	Throat pain	11 (15.71)
	Shortness of breath or difficulty in breathing	9 (12.86)
	General weakness	27 (38.57)
	Bad mental condition	20 (28.57)
	Head pain	10 (14.29)
	Muscle pain	15 (21.43)
	Chest pain	8 (11.43)
	Abdominal pain	5 (7.14)
	Loose stools or diarrhea	14 (20.00)
	Lack of appetite	22 (31.43)
	Nausea	9 (12.86)
	Loss of smell	18 (25.71)
	Loss of taste	22 (31.43)
	Others	5 (7.14)
	Lack of symptoms	21 (30.00)

*: The percentages do not add to 100 because each of the patients could have several symptoms.

**Table 6:** Results of multivariate logistic regression for risk factors predicting symptomatic or severe covid-19.

Feature		Univariate Model				Multivariate Model			
		OR	95% CI		*p*	OR	95% CI		*p*
Age	[years]	1.003	0.96	1.048	0.892	1.057	0.971	1.15	0.199
Gender	Females	1	ref.			1	ref.		
	Males	0.25	0.063	0.987	0.048 *	0.058	0.004	0.78	0.032 *
Comorbid condition: hypertension	No.	1	ref.			1	ref.		
	Yes	0.574	0.155	2.12	0.405	0.526	0.061	4.519	0.558
Comorbidities: Respiratory diseases	No.	1	ref.			1	ref.		
	Yes	1.276	0.169	9.612	0.813	3.768	0.112	127.162	0.46
Comorbidities: Diabetes	No.	1	ref.						
	Yes	1.276	0.169	9.612	0.813				
Comorbidities: Other	No.	1	ref.			1	ref.		
	Yes	0.93	0.285	3.034	0.904	0.139	0.01	1.865	0.136
Place of residence	City	1	ref.			1	ref.		
	Village	3.182	0.856	11.832	0.084	1.113	0.114	10.851	0.926
Social Meetings >50 people 14 days before symptoms/material collection	No.	1	ref.			1	ref.		
	Yes	4.44	0.437	45.151	0.208	9.978	0.516	193.094	0.128
Applying a mask during contact	No contact	1	ref.			1	ref.		
	No.	6.389	1.179	34.624	0.031 *	48.474	0.926	2538.626	0.055
	Yes	5.897	1.89	18.401	0.002 *	37.132	1.708	807.125	0.021 *
Physical distancing more than 2 m during contact	No contact	1	ref.			1	ref.		
	No.	4.533	1.518	13.538	0.007 *	0.39	0.019	7.925	0.54
	Yes	2.667	0.621	11.451	0.187	0.071	0.002	2.097	0.126

* Statistically significance (*p* < 0.05).

**Table 7:** Results of multivariate logistic regression for risk factors predicting scarcely symptomatic, symptomatic, or severe COVID-19.

Feature		Univariate Model				Multivariate Model			
		OR	95% CI		*p*	OR	95% CI		*p*
Age	[years]	0.975	0.929	1.024	0.312	0.978	0.914	1.047	0.525
Gender	Females	1	ref.			1	ref.		
	Males	0.525	0.158	1.741	0.292	0.204	0.033	1.274	0.089
Comorbid condition: hypertension	No	1	ref.			1	ref.		
	Yes	0.762	0.201	2.884	0.689	0.947	0.12	7.451	0.959
Comorbidities: Respiratory diseases	No	1	ref.			1	ref.		
	Yes	0.375	0.049	2.865	0.344	0.24	0.012	4.702	0.347
Comorbidities: Diabetes	No	1	ref.			1	ref.		
	Yes	1.213	0.119	12.403	0.871	1.652	0.02	134.292	0.823
Comorbidities: Other	No	1	ref.			1	ref.		
	Yes	1	0.274	3.656	1	2.944	0.35	24.734	0.32
Place of residence	City	1	ref.			1	ref.		
	Village	1.275	0.307	5.299	0.738	0.335	0.038	2.921	0.322
Applying a mask during contact	No contact	1	ref.			1	ref.		
	No	6.533	0.711	60.05	0.097	5.638	0.127	249.664	0.371
	Yes	5.227	1.577	17.324	0.007 *	4.25	0.306	59.11	0.281
Physical distancing more than 2 m during contact	No contact	1	ref.			1	ref.		
	No	4.792	1.354	16.955	0.015 *	1.608	0.094	27.425	0.743
	Yes	7.5	0.851	66.115	0.07	2.625	0.075	91.712	0.595

* Statistically significance (*p* < 0.05).

## Data Availability

Data are contained within the article. The underlying data will be made available by Authors on demand.
